# Catheter Ablation of Scar-mediated Ventricular Tachycardia: Are Substrate-based Approaches Replacing Mapping?

**DOI:** 10.19102/icrm.2019.100603

**Published:** 2019-06-15

**Authors:** Richard H. Hongo

**Affiliations:** ^1^Atrial Fibrillation and Complex Arrhythmia Center, California Pacific Medical Center, San Francisco, CA, USA

**Keywords:** Catheter ablation, dilated cardiomyopathy, electroanatomic mapping, ischemic heart disease, ventricular tachycardia

## Abstract

Scar-mediated ventricular tachycardia (VT) is a recognized cause of morbidity and mortality in patients with ischemic cardiomyopathy and other cardiomyopathies such as nonischemic cardiomyopathy, arrhythmogenic right ventricular cardiomyopathy, and cardiac sarcoidosis. Implantable cardioverter-defibrillator (ICD) therapy improves survival but does not prevent the onset of recurrent VT or associated morbidity from ICD shocks. While randomized controlled trials have demonstrated advantages of scar-mediated VT ablation in comparison with antiarrhythmic drugs, procedural success has remained overall modest at between 50% and 70%. Standard scar-mediated VT ablation has relied on the use of activation and entrainment mapping during sustained VT to identify critical isthmuses for ablation. Substrate-based approaches have emerged as options to address hemodynamically unstable VT and have focused on identifying electrograms characteristic of critical isthmuses (eg, late potentials, local abnormal ventricular activities, conducting channels) within dense scar during sinus rhythm. Scar homogenization, a more recent approach, relies minimally on mapping and focuses on complete substrate modification. Core isolation, on the other hand, another recent development, relies heavily on mapping to identify regions within scar that are “cores” for arrhythmogenicity and then concentrates ablation to these areas. At this time, scar-mediated VT ablation appears to be at a crossroads wherein evolving substrate-based approaches are exploring whether to rely less or increasingly more on mapping. This review will therefore discuss the evolution of substrate-based, scar-mediated VT ablation and in the process try to answer whether there is still a role for mapping.

## Introduction

Ventricular tachycardia (VT) following myocardial infarction is established as a recognized cause of morbidity and mortality. The risk for cardiac arrest is not limited to affecting patients with ischemic cardiomyopathy, however, and is also observed in the case of other cardiomyopathies such as nonischemic cardiomyopathy (NICM), arrhythmogenic right ventricular (RV) cardiomyopathy (ARVC), and cardiac sarcoidosis. While implantable cardioverter-defibrillator (ICD) therapy has been demonstrated to improve survival in a wide range of cardiomyopathies, it does not directly address the occurrence of recurrent VT nor the associated morbidity from ICD shocks. In the setting of impaired ventricular function, choices for antiarrhythmic therapy are limited by concerns for proarrhythmia. Amiodarone continues to be the primary antiarrhythmic agent utilized for the suppression of scar-mediated VT, but carries with it serious concerns for provoking life-threatening organ toxicity. It is in this setting that catheter ablation has become increasingly utilized in the management of recurrent scar-mediated VT.

Recent randomized controlled trials have examined scar-mediated VT ablation in ischemic cardiomyopathy and have identified an associated decrease in VT storm and ICD shocks.^1–3^ Two of these trials, the VT Ablation in Coronary Heart Disease (VTACH)^1^ and Substrate Mapping and Ablation in Sinus Rhythm to Halt VT (SMASH-VT)^2^ studies, demonstrated a decrease in recurrent VT with prophylactic VT ablation. Society guidelines and consensus statements on the subject of the management of ventricular arrhythmias have supported the performance of catheter ablation even prior to prescribing antiarrhythmic drug therapy in appropriate cases.^4,5^ Furthermore, when freedom from VT is achieved following catheter ablation in patients with ischemic cardiomyopathy, improved survival has been demonstrated.^6,7^

Despite the substantial progress in our understanding of scar-mediated VT and the use of increasingly sophisticated mapping systems and ablation catheters, the success of scar-mediated VT ablation has remained modest and is reported to be between 50% and 70% in most studies. This has led to a reexamination of the standard approach for scar-mediated VT ablation that has traditionally relied on identifying the critical isthmuses for the reentrant VT circuits utilizing activation and entrainment mapping. Substrate-based approaches have evolved over the last 20 years to focus less on the mapping of individual VT circuits and more on substrate modification. Evidence is also emerging that substrate-based approaches offer higher success rates in suppressing VT than the standard approach and that the degree of success is even greater with more complete substrate modification.^8^ This review will examine the evolution of substrate-based, scar-mediated VT ablation and in the process try to answer the question of whether there is still a role for mapping in this regard.

## Standard approach for scar-mediated ventricular tachycardia ablation

Reentry was established early on as the primary mechanism for monomorphic VT in patients with prior myocardial infarction.^9^ While abnormal automaticity accounts for up to 10% of monomorphic VT in ischemic heart disease, reentry is the predominant arrhythmic mechanism and involves slow conduction zones within areas of myocardial scar. These slow conduction zones are composed of surviving myocytes that form strands of viable tissue, sometimes measuring only a single cell thick in diameter, within electrically inert fibrotic tissue that acts to protect the slowed conduction within these channels. Activation within scarred tissue is further characterized by altered ion channel activity, decreased cellular connectivity, and a frequently tortuous course of surviving cells that lead to a “zigzag” pattern of activation.^10^ These protected, slow, abnormally conducting channels within scar are the critical isthmuses for the maintenance of reentry VT and accordingly constitute targets for scar-mediated VT ablation.

### Activation mapping

Identifying the critical isthmus in the standard approach for scar-mediated VT ablation incorporates the performance of cardiac mapping during sustained VT. Activation mapping involves using advanced electroanatomic mapping systems such as CARTO^®^ (Biosense Webster, Diamond Bar, CA, USA); EnSite™ (Abbott Laboratories, Chicago, IL, USA); and RHYTHMIA HDx™ (Boston Scientific, Natick, MA, USA) to create highly detailed, three-dimensional (3D) intracardiac and extracardiac maps that display VT wavefront propagation.^11^ Although low-amplitude slow activation within dense scar is not easily detected by way of conventional activation mapping, the site at the scar border where the reentry circuit exits and starts to reactivate the normal myocardium can be readily observed. Exit site localization facilitates further evaluation of the reentry circuit by utilizing entrainment mapping.

### Entrainment mapping

Entrainment was first described in 1977 by Waldo et al.^12^ as a pacing maneuver performed during sustained arrhythmic episodes that resulted in transient capture without arrhythmia termination and which has since been instrumental in establishing our understanding of the mechanism of reentry. Today, entrainment has become an indispensable mapping technique used to localize the critical isthmuses of reentry tachycardias. In entrainment mapping of scar-mediated VT, pacing within the scar is initiated during sustained VT at a rate slightly faster than that of the VT. When pacing is halted, the interval from the last paced stimulus to the first return of VT activation at the pacing site, also known as the postpacing interval (PPI), is measured. Based on the observation that the PPI is identical to the VT tachycardia cycle length (TCL) when entrainment pacing is performed directly overlying the reentry circuit, the course of the reentry circuit can be traced (eg, a PPI minus the TCL value of less than 30 ms denotes excellent proximity to the circuit).

For a portion of the reentry circuit to be considered a critical isthmus, the site must be bordered on all sides by electrically inert barriers. Consequently, the reentry circuit must activate through this “critical” channel of tissue for arrhythmia maintenance, and any ablation performed at this site would result in arrhythmia termination. Because electrical barriers border the critical isthmus, entrainment pacing at this site results in activation that only propagates antegradely through the protected channel and which emerges from scar in the same fashion as VT activation, creating a paced QRS complex identical to the VT morphology. By assessing the PPI minus the TCL value and the morphology of the paced QRS complex with entrainment mapping, the critical isthmus of the VT circuit and, correspondingly, the site of effective ablation can be identified.

### Challenges and limitations of the standard approach

The primary challenge of the standard approach for scar-mediated VT ablation is the need to perform mapping during sustained VT. Sedation or anesthesia is frequently necessary in prolonged VT ablation procedures but can render VT noninducible and the standard approach unworkable. The standard approach also relies on hemodynamic stability during sustained VT and, when the VT is rapid or unstable, various methods of hemodynamic support such as an intra-aortic balloon pump; Impella^®^ (Abiomed, Danvers, MA, USA); TandemHeart^®^ (LivaNova, London, UK); and extracorporeal membrane oxygenation become necessary to enable mapping. Moreover, multiple VT circuits (ie, median of three but can be more than 10)^13^ are typically found during a single procedure and, since addressing all identifiable VTs and achieving noninducibility have become recognized as necessary endpoints for long-term VT suppression,^14^ this can result in longer procedure times. Sometimes, addressing all inducible VT circuits with the standard approach is not feasible.

Substrate-based approaches initially grew out of necessity when either sustained VT could not be induced or when hemodynamic stability during VT could not be maintained. As the complexity of arrhythmogenic scar has become increasingly recognized, concern has grown that ablation approaches that target only identifiable VT circuits through mapping are not sufficient enough to ensure long-term freedom from VT. Substrate-based approaches are now being attempted for the purpose of modifying the substrate, not only in order to address VT circuits hidden from current mapping techniques but also to proactively eliminate potential VT circuits that may emerge with future scar remodeling.

## Substrate-based strategies for scar-mediated ventricular tachycardia ablation

Voltage mapping has always been instrumental for scar-mediated VT ablation. Defining the extent and pattern of scar has traditionally guided both activation and entrainment mapping. With substrate-based strategies, however, voltage mapping has taken on renewed importance and now includes high-definition mapping that not only defines scar but also looks to find critical isthmuses within scar during sinus rhythm. All substrate-based approaches aim to identify the substrate responsible for VT and then interrupt critical sites of slow conduction necessary for the onset and maintenance of reentry VT.

The majority of critical isthmuses for VT are found within dense scar.^15^ The definition of dense scar is a bipolar electrogram amplitude of less than 0.5 mV, and the cutoff for normal ventricular myocardium is 1.5 mV. By increasing the cutoff for normal myocardium (up to 2.5–3.0 mV), less-dense, often-patchy scar can also become apparent. If minimal scar is found on initial mapping, recreating the voltage map during ventricular pacing can uncover abnormal myocardium through altering the ventricular activation wavefront.^16^ Unipolar voltage mapping has been described as an effective way for assessing abnormal substrate in the epicardium as well as the midmyocardium.^17^ Normal unipolar signal amplitude is defined as that of more than 8.27 mV in the left ventricle and of more than 5.5 mV in the RV.^18,19^

### Linear ablation

Substrate-based ablation for scar-mediated VT started with the creation of linear ablation lesions that typically extended from regions of dense scar out into normal myocardium, frequently transecting the entire length of the scar.^20^ This strategy sought to transect critical isthmuses as they exited scar, and ablation lines were usually placed along borders with exit sites that were confirmed through pacemapping.^21^ Multiple lines could be positioned orthogonally in an effort to transect putative isthmuses traversing scar. A single circumferential ablation line around the entire scar has been utilized by some as a way to theoretically address all potential exits sites and effectively isolate the scar. The main limitation of this strategy was and continues to be, however, the difficulty in achieving block across ablation lines due to ventricular myocardial thickness. It was soon recognized that it was not always necessary to create extensive linear ablations and that targeted ablation within the scar also resulted in successful elimination of VT.

### Late-potential ablation

From early on, fragmented electrograms, typically characterized as low-amplitude, high-frequency continuous signals, have been correlated with slow conduction.^22^ Fragmented signals that occur distinctly separate from local ventricular activation (“isolated”) and which are delayed at least 40 ms to 50 ms after the surface QRS complex (“late”) are more specific types of fragmentation that correspond with critical isthmuses for VT.^23^ A longer delay to late potential (ie, a longer QRS–late potential interval) corresponds with a critical isthmus deeper within the scarring and farther from the exit site.^24^ The strategy of late-potential ablation seeks to eliminate all identifiable late potentials and is highly dependent on the ability to locate all necessary late potentials. Ventricular pacing can make late potentials more evident by altering the ventricular activation wavefront. Success of late-potential ablation also depends on the ability to effectively eliminate the late potentials. This is particularly a challenge when late potentials involve thicker papillary muscles within the scarred region.

### Local abnormal ventricular activity ablation

Local abnormal ventricular activities (LAVAs) are described as sharp, high-frequency ventricular potentials, possibly but not necessarily low-amplitude in nature, that occur during (but distinct from) or after the far-field ventricular electrogram in sinus rhythm.^25^ LAVAs differ from late potentials in that they are not necessarily late and not always low in amplitude. The presence of LAVAs is felt to identify abnormal tissue that is poorly coupled to normal myocardium and is confirmed by demonstrating delay of the local high-frequency electrogram from the far-field ventricular electrogram using pacing maneuvers, commonly progressively earlier ventricular extrastimulation. Late LAVAs are essentially late potentials. By using LAVAs to describe arrhythmogenic substrate instead of late potentials, critical ablation sites can be localized in regions that are less likely to demonstrate delayed potentials, such as scar borders or regions of the ventricle that typically activate early, such as the septum.^26^ Similar to late-potential ablation, the strategy of LAVA ablation is dependent on the ability to identify and then effectively eliminate all necessary LAVA signals.

### Scar dechanneling

Conducting channels have been found within scar in as many as 75% of patients, and elimination of these conducting channels has resulted in the noninducibility of and clinical freedom from VT.^27^ Different methods of identifying channels have been described. High-output pacing (defined as 10 mA with a 2-ms pulse width) has been employed to distinguish unexcitable scar from conducting channels.^28^ With this method of pacemapping, Stimulus–QRS delay can tract the course of the critical isthmus as well as localize the exit site at the scar border. High-density mapping coupled with voltage criteria adjustment (progressively lower cutoffs for dense scar to normal tissue, ie, 0.50–0.51, 0.40–0.41, 0.30–0.31, 0.20–0.21, and 0.10–0.11 mV) have been effective in uncovering critical isthmuses.^27^ The earliest activation within these channels is targeted first, as this has the potential to electrically silence the channel downstream. The primary challenge of this approach is defining channels that can be underdetected depending on mapping density and because of intramural and epicardial channels.

### Scar homogenization

The strategy of scar homogenization that seeks to eliminate all detectible electrograms within the scar region may represent the purest expression of substrate-based ablation to date. In its most complete form, this strategy involves both endocardial and epicardial homogenization. Apart from voltage mapping to define the extent of scarring and activation mapping to confirm that the VT is associated with identified scars, further mapping is generally not performed and ablation is empirically extended throughout the scar. Along with the total loss of electrograms within the scar and noninducibility, noncapture of the scar with high-output pacing (defined as 20 mA with a 10-ms pulse width) has been utilized as an endpoint.^29^ While freedom from VT after scar homogenization is lower in patients with NICM versus ischemic cardiomyopathy, scar homogenization is still associated with higher freedom from VT after 14 months than standard VT ablation in NICM patients (63.9% versus 38.6%; p = 0.031).^30^ A recent meta-analysis evaluated the long-term outcomes of substrate modification versus standard ablation of VT and found that the former was associated with a decreased composite of recurrent VT and mortality [risk ratio (RR): 0.57, 95% confidence interval (CI): 0.40–0.81]. Moreover, complete substrate modification as compared with incomplete substrate modification was associated with lower VT recurrence (RR: 0.39, 95% CI: 0.27–0.58).^8^ While there are concerns that, with the performance of complete substrate modification, nonarrhythmogenic regions undergo unnecessary ablation, conceptually, this strategy is likely able to address arrhythmogenic substrate not yet identifiable using current mapping techniques. Questions remain regarding the optimal extent of ablation at each site, indications for epicardial homogenization, and appropriate procedural endpoints. It is also unclear as to whether there are potential long-term effects of extensive ventricular ablation such as a decline in overall systolic and diastolic ventricular function or an increase in postprocedural intraventricular thrombosis risk that could warrant long-term anticoagulation.

### Core isolation

With this more recent strategy for scar-mediated VT ablation, mapping is reintroduced as an important part of what is still a substrate-based approach.^31^ Critical or “core” VT circuit elements are first identified via detailed electrophysiological characterization. High-density voltage mapping defines the extent of scar but is also assessed for evidence of conducting channels within dense scar. Pacemapping within the scar verifies the presence of channels, while pacemapping along the scar border localizes putative exits sites. Fractionated and late potentials are identified and annotated. Programmed stimulation is then performed and stable sustained monomorphic VT is ablated with guidance from activation and entrainment mapping. Next, circumferential ablation is performed around a portion of the scar that includes potential channels, putative exit sites, and fractionated and late potentials. The endpoints are the achievement of electrical isolation of this core region with failure to capture with high-output pacing (20 mA with 2-ms pulse width) and noninducibility of VT. Reinforcing lesions are placed within the isolated region targeting previously annotated sites of fractionated or late potentials. It has been noted that, to achieve isolation of the core, ablation must extend to electrical barriers and the presence of inexcitable intramural scar may be necessary for isolation, regardless of whether ablation is being performed from endocardial or epicardial surfaces. Protracted ablation applications have been required (at times with durations of more than 90 seconds) in order to achieve what has been described to be necessary impedance drops of 12 Ω to 15 Ω.^31^

## Other considerations for scar-mediated ventricular tachycardia ablation

### Nonischemic cardiomyopathy

Whereas scar in ischemic heart disease develops from the subendocardium to epicardium according to myocardial blood supply, scar in NICM is less predictable and has a predilection for the midmyocardium and epicardium. Although scar-mediated VT ablation has mostly been evaluated in ischemic cardiomyopathy, activation, entrainment, and pacemapping techniques have also been found to be effective in NICM.^32^ Generally, however, the success of VT ablation in NICM has been less than that in ischemic cardiomyopathy. When epicardial ablation is included as an adjuvant to endocardial ablation, long-term (60 months) freedom from VT can be as high as 69% in experienced centers.^33^ Delayed-enhanced cardiac magnetic resonance imaging (MRI) can help with identifying scarring in NICM and augmenting the ability to pinpoint successful sites for ablation.

### Arrhythmogenic right ventricular cardiomyopathy

**Figure 1:**
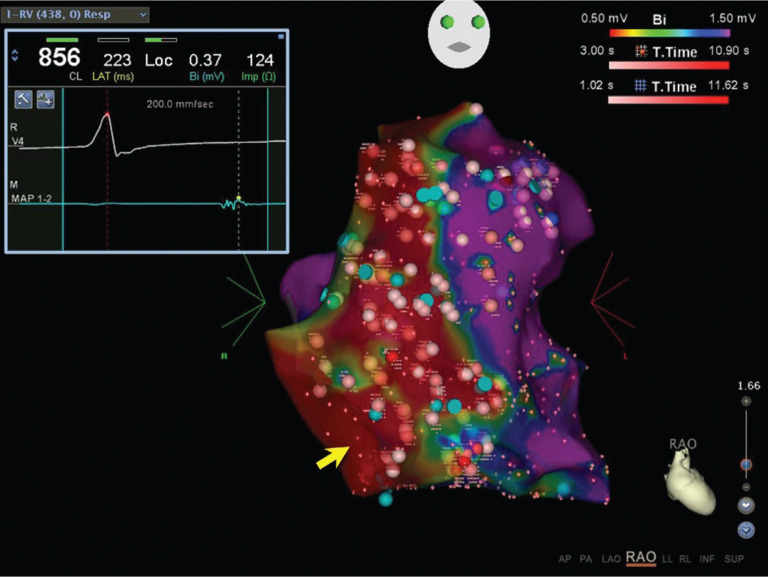
3D electroanatomic RV endocardial voltage map of a patient with ARVC created using CARTO^®^ (Biosense Webster, Diamond Bar, CA, USA). Dense scar is defined as that less than 0.5 mV and is depicted in red. Late potentials are marked with turquoise-colored tags. Inset electrogram from the ablation catheter distal tip (MAP 1–2) displays a markedly delayed late potential at a site within dense scar (yellow arrow). High-output (45–50 W), open-irrigated ablation targeted all late potentials and any detectable signal within the dense scar and are marked with red tags (shades of red depict ablation duration).

**Figure 2:**
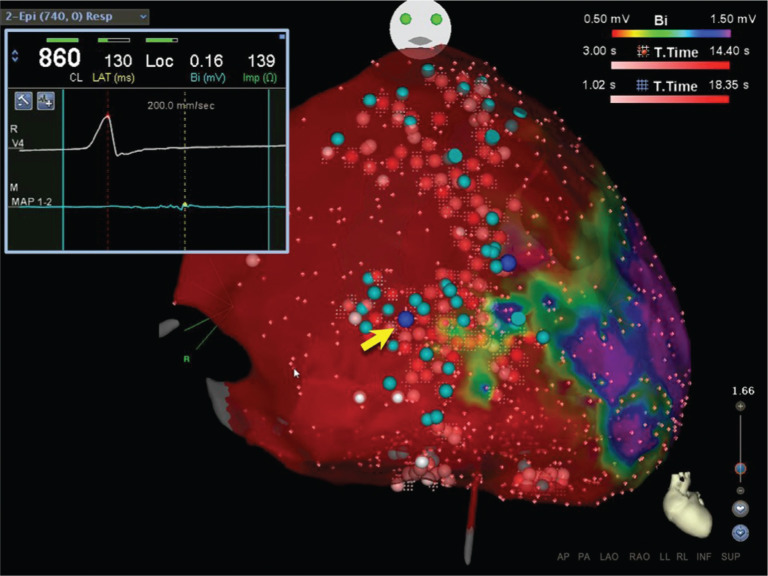
3D electroanatomic RV epicardial voltage map created using CARTO^®^ (Biosense Webster, Diamond Bar, CA, USA) of the same patient with ARVC described in **Figure 1**. Dense scar is defined as that less than 0.5 mV and is depicted in red. Late potentials are marked with turquoise-colored tags and reveal a branching pattern of conducting channels within epicardial scar. Inset electrogram from the ablation catheter distal tip (MAP 1–2) displays a continuous, low-amplitude signal within a conducting channel (yellow arrow). Dechanneling was performed using high-output (45–50 W), open-irrigated ablation and sites are marked with red tags (shades of red depict ablation duration).

**Figure 3:**
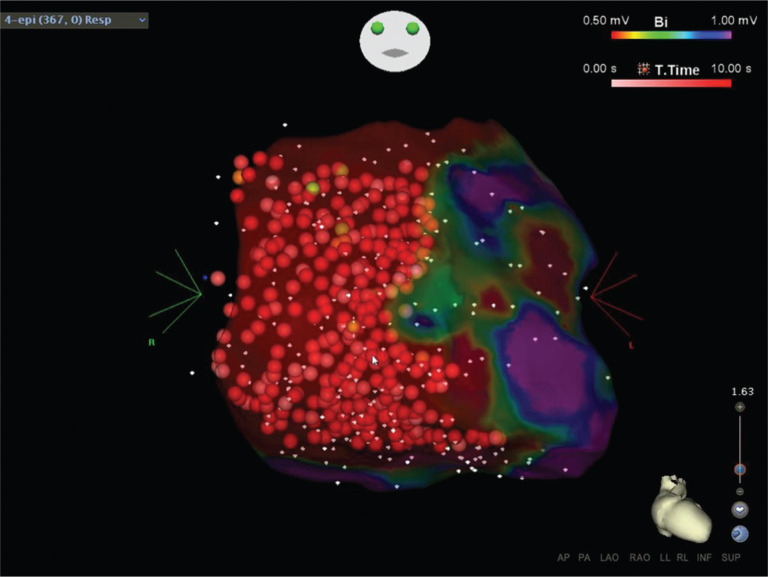
3D electroanatomic RV epicardial voltage map created using CARTO^®^ (Biosense Webster, Diamond Bar, CA, USA) at one year later of the same patient with ARVC described in **Figure 1**. Dense scar is defined as that less than 0.5 mV and is depicted in red. The presentation of recurrent monomorphic VT at one year after the first ablation is an example of either the progression of ARVC or the limitations of less-complete, substrate-based ablation approaches. Endocardial (not shown here) and epicardial scar homogenization using high-output (45–50 W), open-irrigated ablation was performed and sites are marked with red tags (shades of red depict ablation duration).

Genetic mutations that primarily compromise desmosomal integrity are known to result in the detachment of myocytes during increased mechanical stress in ARVC and in progressive fibrofatty replacement of myocardium. This progressive process predominately affects the RV and characteristically initiates from the epicardium. A markedly delayed, long, continuous, and highly fractionated signal is a hallmark finding of ARVC and the elimination of these signals has become an important endpoint for ablation.^34^ Incorporation of epicardial ablation has improved ablation success in ARVC and, in one study, a combined endocardial–epicardial substrate-based approach promoted a three-year freedom-from-VT prevalence of 84.6% as compared with a prevalence of 52.2% achieved with an endocardial-only approach.^35^ The durability of successful ablation is unclear, however, as ARVC is a progressive disease that can create new arrhythmogenic scar over time **(Figures 1–3)**.

### Cardiac sarcoidosis

Sarcoidosis is an inflammatory condition of unknown etiology that results in noncaseating granulomas most commonly affecting the lungs, but cardiac involvement manifests in 5% of patients with the disease. In addition to heart block and heart failure, unheralded sudden cardiac death from VT is a rare but serious problem. Aside from general ICD indications for NICM, ICD therapy is also considered for use in patients with pacemaker indication, a left ventricular ejection fraction of less than 50%, an RV ejection fraction of less than 40%, and delayed-enhancement findings on cardiac MRI.^36^ Treatment usually involves immunosuppressive therapy with corticosteroids. In a large study of patients presenting for catheter ablation, a propensity for confluent RV scarring was described in contrast to scarring in the left ventricle that tended to be patchy with a predilection for the septum.^37^ The indications for VT ablation in cardiac sarcoidosis are still largely unclear, but, when ablation was performed, the finding of a failure to abolish all inducible VTs was common,^37^ the need for repeat ablation was high, and the rate of long-term (two-year) freedom from VT after multiple procedures was as low as 55%.^38^

### Epicardial ablation

Since percutaneous pericardial access for catheter ablation was first described by Sosa et al.,^39^ epicardial VT ablation has come to represent more than 10% of VT ablation procedures and has become important especially in NICM as well as critical for success in ARVC and cardiac sarcoidosis. The choice between using a posterior or anterior approach is typically based on the anticipated region of ablation. In patients with prior cardiac surgery, the posterior approach may be preferable because of the high occurrence of anterior adhesions related to surgical pericardiotomy. Surgical subxiphoid pericardial access can facilitate better exposure of bypass grafts and safe dissection of adhesions.^40^ While reported complication rates for percutaneous pericardial access vary widely, excessive pericardial bleeding (> 80 mL) from inadvertent injury to the RV or coronary arteries is considered to be the most common complication, with an incidence usually reported as less than 10%. The “needle-in-needle” approach—which involves advancing a long, 21-gauge micropuncture needle within a shorter 18-gauge needle—has been associated with a significantly lower incidence of large pericardial effusions in comparison with approaches using conventional large bore needles such as the Tuohy or Pajunk^®^ options (Pajunk Medical Systems, Norcross, GA, USA) (0.9% versus 8.1%; p < 0.001).^41^

Careful assessment of the 12-lead electrocardiogram during VT can help to determine if the exit site is epicardial in nature. Criteria that have been found to correspond with epicardial VT (≥ 95% specificity) include a Q-wave in lead I, no Q-waves in inferior leads, a pseudo-delta wave of greater than or equal to 75 ms, and a maximum deflection index of greater than or equal to 0.59. Combining these criteria in a four-step algorithm demonstrated high sensitivity (96%) and specificity (93%) for identifying epicardial activation.^42^ Challenges during epicardial ablation include epicardial fat that can interfere with effective power delivery and the course of phrenic nerves and coronary arteries, which can limit the extent of ablation.

### Deep intramural ablation

While irrigated ablation catheters have facilitated deeper energy delivery, deep intramural ablations have continued to be a challenge, especially when involving the septum. Various techniques for deep intramural ablation have been utilized including transcoronary ethanol ablation,^43^ similar to the alcohol septal ablation procedure performed in hypertrophic cardiomyopathy. Needle-irrigated ablation has been described as where an extendable/retractable 27-gauge nitinol needle is advanced 7 mm to 9 mm into myocardium for effective deep intramural mapping and ablation.^44^ The use of two separate ablation catheters placed on either side of a thick portion of myocardium has also been reported, either in the context of performing simultaneous unipolar ablations (using two separate radiofrequency generators)^45^ or of true bipolar ablation (using a custom-made connector to connect the second ablation catheter to the indifferent electrode port of the generator).^46^

## Future innovations

Ripple mapping with the CARTO^®^ 3 system (Biosense Webster, Diamond Bar, CA, USA) is a more sophisticated 3D display of the propagating wavefront, allowing slowly conducting channels within ischemic scar to be better visualized, making the process of identifying critical isthmuses within scar with activation mapping more feasible.^47^ Electrocardiographic imaging using a 252-electrode vest is being evaluated as a noninvasive 3D mapping option that can facilitate the preprocedural mapping of VT.^48^ While delayed-enhanced cardiac MRI has been shown to aid in identifying scarring not apparent with electroanatomic mapping,^49^ inadequate spatial resolution for identifying critical isthmuses has remained a major limitation of current technology. Real-time MRI guidance may better facilitate VT ablation and continues to be evaluated. Stereotactic radiotherapy has been reported as a novel, noninvasive cardiac ablation method that safely decreases VT burden and which may lead the way to newer paradigms beyond catheter ablation in the management of scar-mediated VT.^50^

## Conclusions

Substrate-based approaches for scar-mediated VT ablation emerged beginning about 20 years ago, initially as a way to address hemodynamically unstable VT. Early substrate-based ablation approaches focused on targeting electrocardiographic markers of critical isthmuses within scar, such as late potentials, LAVAs, and conducting channels. However, with increasing recognition of the complexity of arrhythmogenic scar, more complete forms of substrate modification have since been developed. Scar homogenization electrically silences the entire scar with closely spaced ablations, both endocardially and epicardially, with minimal reliance on mapping, and has demonstrated improved freedom from VT as compared with the standard approach and other, less-complete forms of substrate modification. Unanswered questions regarding complete substrate modification involve long-term effects of extensive ablation on overall systolic and diastolic ventricular function, postprocedural intraventricular thrombosis risk, and appropriate procedural endpoints. Core isolation is a more recent substrate-based approach that seeks to concentrate ablation on scarred regions determined to be “cores” for arrhythmogenicity and potentially spare nonarrhythmogenic regions from ablation, mitigating some of the concerns for overextensive ablation. This approach relies on the accurate identification of core regions using activation, entrainment, and high-definition scar mapping. Based on this review, at this time, scar-mediated VT ablation is far from abandoning mapping, and appears to be at a crossroads wherein evolving substrate-based approaches are exploring whether to rely less or increasingly more on mapping.
